# Reduction in total leukocytes in malaria patients compared to febrile controls: A systematic review and meta-analysis

**DOI:** 10.1371/journal.pone.0233913

**Published:** 2020-06-23

**Authors:** Manas Kotepui, Kwuntida Uthaisar Kotepui, Giovanni D. Milanez, Frederick R. Masangkay

**Affiliations:** 1 Medical Technology, School of Allied Health Sciences, Walailak University, Thasala, Nakhon Si Thammarat, Thailand; 2 Department of Medical Technology, Institute of Arts and Sciences, Far Eastern University, Manila, Philippines; Instituto Rene Rachou, BRAZIL

## Abstract

**Background:**

Leukocyte alterations are a common hematological alteration among malaria patients.

**Objectives:**

This systematic review and meta-analysis aimed to provide data and evidence comparing alterations in total leukocyte counts in malaria patients compared to febrile/healthy subjects at baseline before treatment. A systematic review was conducted by following the Preferred Reporting Items for Systematic Reviews and Meta-Analyses (PRISMA) statement for reporting systematic reviews and meta-analyses.

**Data sources:**

Web of Science (ISI), Scopus, and Medline.

**Study eligibility criteria, participants, and interventions:**

All published articles reporting a total leukocyte count of patients infected with malaria, non-malaria (febrile or healthy group) at baseline before treatment before August 27, 2019, were retrieved, and data were extracted by two main reviewers independently.

**Study appraisal and synthesis methods:**

We used a forest plot, heterogeneity test (Cochran’s Q), and the degree of heterogeneity (I^2^) to test whether the included studies were heterogeneous. The quality of the included studies was determined by a quality assessment guide based on the quality assessment tool developed by the Newcastle-Ottawa Scale (NOS). Cochran’s Q (Chi-square) and Moran's I^2^ were used to evaluate heterogeneity. Meta-regression using STATA software was conducted to find the source of heterogeneity. A funnel plot with Egger’s test was used to examine the significance of publication bias among the included studies. The mean differences were estimated using a random-effects model.

**Results:**

Out of the 2,261 articles screened, 29 articles were included in this systematic review and meta-analysis. The heterogeneity test indicated that there was heterogeneity among the included studies with no publication bias. The meta-analysis demonstrated that the total leukocyte count was significantly lower in patients with malaria (n = 4,619) than in those without malaria (n = 10,056) (Z = 4.0, P-value < 0.00001, mean difference = -1.38, 95% CI = -2.06-(-0.71)). Leukocyte differential alterations, low lymphocyte counts (P-value <0.0001, mean difference = -1.03, 95% CI = -1.53-(-0.53)) and a high NL ratio were found in the malaria group (n = 1,579) compared to the non-malaria group (n = 4,991) (P-value <0.0001, mean difference = 0.6, 95% CI = 0.32–0.88). The subgroup analysis indicated that there was a significantly lower total leukocyte count in the malaria group (n = 3,545) than in the febrile group (n = 8,947) (Z = 1.33, P-value < 0.0001, mean difference = -1.76, 95% CI = -2.56-(-0.96)), but no significant difference was found between the malaria group (n = 1,232) and the healthy group (n = 1,679) (P-value > 0.05).

**Limitations:**

As the specific diagnoses in the febrile groups were not reported in the included studies so that the results of the present study need to be carefully interpreted.

**Conclusions and implications of key findings:**

This systematic review demonstrated that the total leukocyte count was affected by malarial infection at baseline despite the heterogeneity of the included studies. Future work must aim to understand the treatment-related total leukocyte reduction during follow-up or post-treatment outcomes in malaria-endemic settings.

## Introduction

Malaria is a major public health problem worldwide, especially in sub-Saharan Africa, with estimated 228 million cases and 405,000 deaths worldwide in 2018 [1]. The clinical manifestations of malaria patients can be divided into uncomplicated malaria and severe malaria. Severe malaria is characterized by the presence of one of the following: bleeding or disseminated intravascular coagulation (DIC), metabolic acidosis, prostration, severe anemia, hypoglycemia, shock, jaundice, impaired consciousness, multiple convulsions, acute kidney injury, or pulmonary edema [2]. Uncomplicated malaria is characterized by nonspecific symptoms, with fever as a hallmark and other nonspecific signs, such as malaise, anorexia, headache, myalgia, nausea, vomiting or chills [3]. Laboratory findings of uncomplicated and severe malaria show some degree of anemia and thrombocytopenia [4, 5–7, 8, 9], which are the two most recognized laboratory findings among most literature reviews. However, the overall understanding of leukocyte alterations in uncomplicated and severe malaria is still incomplete, and this is the first gap addressed in the present study.

Leukocyte alterations are a common hematological alteration among malaria patients [7, 10–18]. Previous studies have described leukopenia during malarial infection [7, 11–15, 18]. However, some studies observed leukocytosis during malarial infection [10, 16, 17]. A previous study by Zahorec et al. introduced the neutrophil-lymphocyte ratio (NLR) as a better indicator of systemic inflammation and stress than C-reactive protein (CRP) level [19]. Our previous study also demonstrated that neutrophil and lymphocyte counts were the most important leukocytic changes associated with malaria infection as NLR in malaria infected patients was higher in comparison to non-malaria infected patients [13]. To date, there have been no systematic reviews or meta-analyses focusing on leukocyte alterations during malarial infection. The association between leukocyte counts during malarial infection is limited. Previous studies demonstrated that during early malarial infection, the leukocyte count decrease was related to fever outcomes [20, 21]. The alteration of leukocyte counts in combination with routine malaria diagnosis, such as microscopy techniques, in malaria-endemic areas may prove beneficial for laboratory technicians or physicians, especially in patients with very low parasitemia. Alterations of leukocyte counts might be used in combination with other markers to help diagnose malaria and could be useful for the management of malarial patients.

The second gap in previous studies is that most of the studies reported a significant difference in hematological parameters in febrile patients who were negative for the malaria parasite as a non-malaria group. However, these patients might have other infections and therefore do not represent a healthy population. These bacterial or viral infections might affect hematological variables in different ways. This systematic review and meta-analysis aimed to provide data and evidence comparing total leukocyte alterations among malaria patients and febrile/healthy subjects at baseline before treatment.

## Materials and methods

A systematic review was conducted by following the Preferred Reporting Items for Systematic Reviews and Meta-Analyses (PRISMA) statement for reporting systematic reviews and meta-analyses of studies that evaluate healthcare interventions [22] (see S1 Checklist).

### Definitions

The malaria group included patients who were infected with at least one of five *Plasmodium* species, which included *P*. *falciparum*, *P*. *vivax*, *P*. *ovale*, *P*. *malariae*, and *P*. *knowlesi*. The febrile group included patients who were recruited for the assessment of malaria parasites, but no malaria parasites were found. The healthy group was the group of patients who were assessed as healthy in the same area of study.

### Eligibility criteria

Searches for this study were limited to human studies but were not limited by year, country or language. Only original research studies with quantitative analysis were considered, thereby excluding animal studies, clinical drug trials, in vitro and in vivo studies, reviews, systematic reviews, short reports, letters to the editor, quizzes, and articles for which the full text was unavailable. Further, studies which involve the analysis of baseline leukocyte count for both malaria groups and non-malaria groups, and. studies of enrolled patients with malarial infection and non-malaria patients (febrile or healthy) with a report on the total leukocyte count at baseline before treatment were included. Studies involving patients with hematological diseases (sickle cell anemia, thalassemia, and hemoglobinopathies), hematological malignancies (lymphoma, leukemia, and multiple myeloma), chronic liver disease (hepatitis B and C), or other diseases/conditions such as human immunodeficiency virus infection, acquired immune deficiency syndrome (HIV/AIDS), organ transplantation, pregnant women, or were exhibiting mixed infections were excluded from this study.

### Search strategy

Published studies were identified using keywords in combination with truncations. AND with OR was used to combine terms “(malaria OR plasmodium) AND (leukocyte OR white blood cell)” (see S1 Table). The searches for articles from all three databases started on 27 August 2019 and finished on 28 August 2019. The searches were conducted in the following three main research databases: MEDLINE (1947–2019, 27 August), SCOPUS (1921–2019, 27 August), and ISI Web of Science (2002–2019, 27 August). The papers were imported into EndNote X9 (Thomson Reuters, USA) for reference management. Two main reviewers (MK and KK) independently examined all papers and performed the study selection. The first step of reviewing the papers was the identification of relevant articles based on titles and abstracts. If a paper was potentially related or if it was unclear if it was related, a full-text review of the paper was performed before a decision was made to include or exclude it from this study. The second step of reviewing the papers was reading the text of the articles, which was conducted by each reviewer independently. For discordances between the two reviewers regarding full article reviews, a third reviewer participated and decided whether the study should be included or excluded.

### Data extraction

Data extraction was conducted for each selected article, and the following data were extracted: author, references, study area, year of study, mean age, age range, sex ratio, type of *Plasmodium* sp., and severe complications. The detection of malaria parasites were based on any of these methods: rapid diagnostic test (RDT), microscopy, polymerase chain reaction (PCR), or any test combination. The number of participants in malaria and non-malaria groups, characteristics of controls, and diagnostic techniques were also extracted. Extracted data were entered into an Excel sheet.

### Quality of included studies

Quality assessment was performed with the quality assessment tool developed by the Newcastle-Ottawa Scale (NOS) for assessing the quality of nonrandomized studies in meta-analyses [23]. The quality assessment tool was used to evaluate the validity of the included studies, which is also shown in Table 2.

### Meta-analysis

A meta-analysis was conducted using Review Manager (RevMan) 5.3 software (Version 5.3, London, UK). Heterogeneity was assessed using Cochran’s Q (Chi-square) and Moran's I^2^. In cases where heterogeneity existed, meta-regression was performed using STATA software (StataCorp, USA), and subgroup analyses were conducted to explore the source(s) of heterogeneity. For studies that reported the median and range, we estimated the mean and standard deviation according to the method devised by Hozo et al. [24]. Missing standard deviations were calculated by the imputation of average standard deviations borrowed from other studies according to the method devised by Furukawa et al. [25]. Mean differences and the 95% confidence interval were the effect measures for the mean differences in the total leukocyte count among malaria and non-malaria groups and were calculated by using the generic inverse variance method and random-effects model. Moreover, mean differences in leukocyte differential count for neutrophils, lymphocytes and the neutrophil/lymphocyte ratio (NL ratio) were also analyzed.

### Assessment of publication bias

Publication bias was evaluated using a funnel plot. Egger’s test was used to test for funnel plot asymmetry.

## Results

### General characteristics of included studies

**Fig 1:**
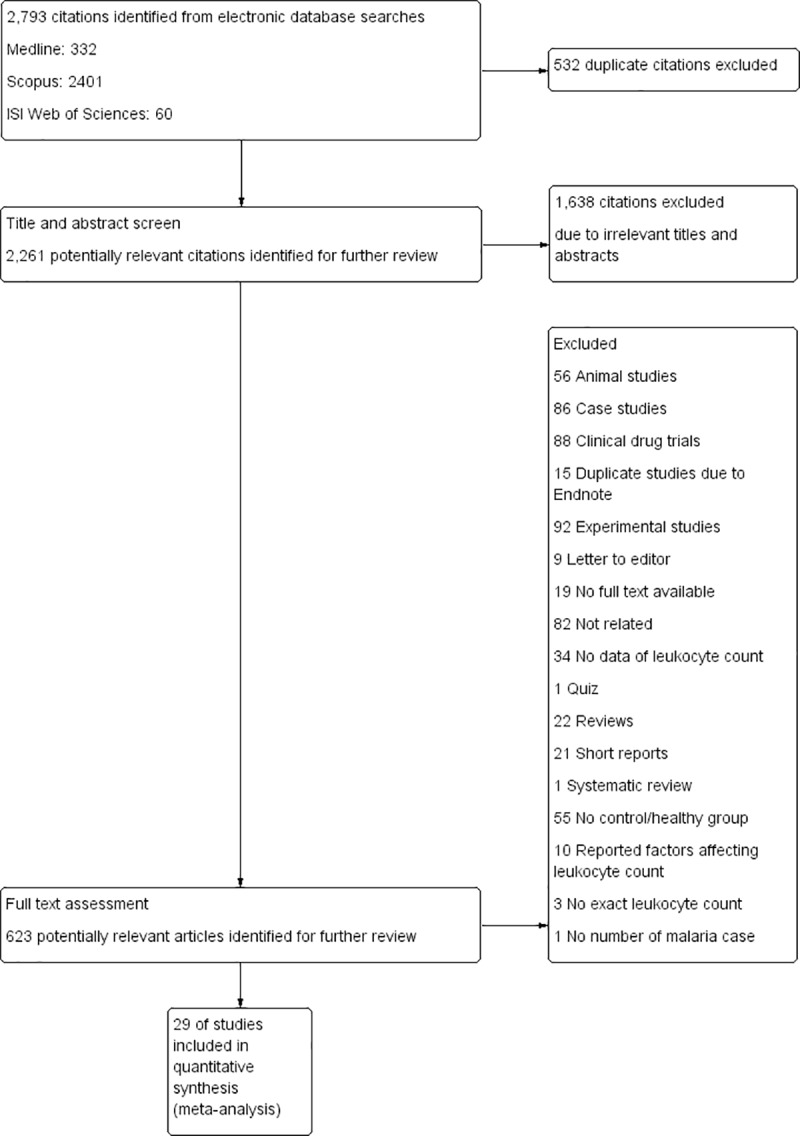
PRISMA diagram. Flow chart for study selection.

A total of 2,261 potentially relevant articles were identified for this systematic review after duplicate citations were removed. After reviewing the title and abstract, 623 articles were selected for the full text review. Among the 623 articles, 594 were removed because they did not report the leukocyte count or because of any of the inclusion and exclusion criteria in this study. Out of 2,261 potentially relevant articles, 29 met the inclusion criteria and were subsequently included in this review (Table 1). The majority of the studies were conducted in African countries (15 of 29 studies). Seven studies were conducted in Asian countries, and six studies were conducted in South America. Eight of the articles from five African countries–Ghana [26], Gabon [7, 27], Nigeria [28, 29], Cameroon [9, 30], and Sudan [31]–reported only *P*. *falciparum* infection, while seven of the articles in Brazil [15, 32, 33], Venezuela [34], Republic of Korea [4], Turkey [35], and Ghana [36] reported only *P*. *vivax* infection. *P*. *ovale* infection was described only in three studies conducted in France [37], Thailand [6], and South Africa [38]. *P*. *malariae* infection was described only in two studies conducted in Thailand [6] and Nigeria [8]. Mixed infections of *P*. *falciparum* and *P*. *vivax* were found only in Ethiopia [5], Thailand [6], Brazil [39], and India [40], whereas mixed infections of *P*. *vivax*/*P*. *malariae* and *P*. *falciparum*/*P*. *ovale* were found in Nigeria [8] and South Africa [38], respectively. The mean age of the participants was 23±13.1 years, while the sex ratio of males/females was 1.15:1. All articles were published between 1997 and 2019 (Fig 1). Six studies reported 224 cases of severe malaria in their publications [7, 27, 30, 31, 36, 38]. Most of the severe complications in the studies were severe anemia (21.9%, 49/224), cerebral malaria (13.8%, 31/224), hyperparasitemia (9.8%, 22/224), repeated convulsions (8%, 18/224), more than one complication (3.6%, 8/224), hypotension (3.1%, 7/224), jaundice (2.7%, 6/224), hypoglycemia (1.8%, 4/224), and prostration (0.4%, 1/224). The non-malaria groups were divided into febrile and healthy groups based on the descriptions by the authors. The febrile group included patients who were suspected of having malaria, but blood parasitemia was negative by any of these techniques, including microscopy, rapid diagnostic tests (RDTs), and polymerase chain reaction (PCR). The healthy group included healthy individuals in the same endemic area as the malarial patients in their studies.

**Table 1:** Characteristics of the included studies.

References	Study area (years of the survey)	Mean age of the malaria group (years)	Mean age of the non-malaria (years)	Male sex	*Plasmodium* sp.	Total leukocyte count (10^3^/μL) (mean ± SD)	Severity	Source of leukocyte count	All participants	Cases of P.f	Cases of P.v	Cases of P.o	Cases of P.m	All cases	Non-malaria	Characteristics of non-malaria
Adam et al., 2017 [41]	Sudan (2014–2015)	20.7 ±19.6	20±19	All groups = 50%; Malaria = 50%; Non-malaria = 50%	*P*. *falciparum* and *P*. *vivax*	Mean leukocyte count in malaria 6.7± 0.9; -P.f 6.6±0.9; -P.v 6.9±1.3; Mean leukocyte count in non-malaria 8.1± 1.4	UM	Sysmex XN-9000; Hyogo, Japan	324	107	55			162	162	Febrile
Ansart et al., 2010 [37]	France (2002–2003)	1.1–55	1.1–42	All groups = 57%; Malaria = 57%; Non-malaria = 57%	*P*. *falciparum*, *P*. *vivax*, and *P*. *ovale*	Mean leukocyte count in malaria 9.25; Mean leukocyte count in non-malaria 11.2	NA	NA	272	36	14	4		34	218	Febrile
Anwar et al., 2016 [42]	Pakistan (2015–2016)	15–20	21–60	All groups = 100%; Malaria = 100%; Non-malaria = 100%	Did not define species	Mean leukocyte count in malaria 5.03±13.8; Mean leukocyte count in non-malaria 9.5±13.8	NA	NA	65	Species not defined 60				60	5	Healthy
Awoke N and Arota A, 2019 [5]	Ethiopia (2016)	27.6	27.6	All groups = 70%; Malaria = 70%; Non-malaria = 70%	*P*. *falciparum*, *P*. *vivax*, *and* mixed infection (4 cases)	Mean leukocyte count in malaria 5.3±2.2 (including mixed infections); Mean neutrophil count 3.52±1.8; Mean lymphocyte count 1.17±0.65; NL ratio = 3±2.77 Mean leukocyte count in non-malaria 5.8±1.8; Mean neutrophil count 3.30±1.4; Mean lymphocyte count 1.78±0.58; NL ratio = 1.85±2.41	NA	CELL-DYN 1800	340	105	61	-		166	170	Febrile
Chaves et al., 2016 [15]	Brazil (NA)	35.4 ±14.3	27.1 ±7.4	All groups = 54.5% Malaria = 45% Non-malaria = 64%	*P*. *vivax*	Median leukocyte count in malaria 5.7 (4.0–7.3); Mean neutrophil count 3.45±0.89; Mean lymphocyte count 1.28±0.38; NL ratio = 2.7±2.34 Median leukocyte count in non-malaria 6.7 (6.1–7.8); Mean neutrophil count 4.05±0.53; Mean lymphocyte count 2.05±0.35; NL ratio = 1.98±1.51	UM	Sysmex KX-21 N®	56	-	36	-		36	20	Healthy
Erhart et al., 2004 [6]	Thailand (2001)	All groups = 28		All groups = 63%	*P*. *falciparum*, *P*. *vivax*, *P*. *malariae*, *P*. *ovale*, and mixed infections (23 cases)	Mean leukocyte count in malaria 6.4; -P.f 6.3; -P.v 6.6 (excluding mixed infections) Mean leukocyte count in non-malaria 8.4	UM	Beckman-Coulter, Inc, Fullerton, CA	2149	414	646	2	15	1060	979	Febrile
Frimpong et al., 2018 [26]	Ghana (NA)	6.5 (4.7–8)	9 (8–11)	All groups = 50%; Malaria = 47%; Non-malaria = 53%	*P*. *falciparum*	Mean leukocyte count in malaria 6.4 (3.2–9.0); Median leukocyte count in non-malaria 7 (5.7–8.0)	UM	NA	57	40				40	17	Healthy
Goncalves et al., 2010 [39]	Brazil (NA)	35 (23.3–42.3)	32 (28–42)	All groups = 57.1%; Malaria = 55.5%; Non-malaria = 58.7%	*P*. *falciparum*, *P*. *vivax*, *and* mixed infections (14 cases)	Mean leukocyte count in malaria 5.6±0.7; -P.f 5.3±0.8; -P.v 5.7±0.6 (excluding mixed infections); Mean leukocyte count in non-malaria 7.8±1.1	UM	ABX Micro 60, Horiba, Montpellier, France	80	19	43			62	18	Healthy
González et al., 2009 [34]	Venezuela (NA)	3–67	NA	All groups = 64.4; Malaria = 64.4%; Non-malaria = NA	*P*. *vivax*	Mean leukocyte count in malaria 7,01±2,34; Mean neutrophil count 4.96±2.0; Mean lymphocyte count 2.98±1.28; NL ratio = 1.66±1.56 Mean leukocyte count in non-malaria 8,01 ± 2,03; Mean neutrophil count 4.27±1.75 Mean lymphocyte count 4.89±1.41; NL ratio = 0.96±1.24	NA	NA	69		59			59	30	Healthy
Hänscheid et al., 2008 [7]	Gabon (2003–2004)	3.7	0.6	All cases = 46%	*P*. *falciparum*	Mean leukocyte count in malaria 8.7±9.6; Mean leukocyte count in severe malaria 10±3.92; Mean leukocyte count in uncomplicated malaria 8.1±2.6; Mean neutrophil count 3.8±1.49; Mean lymphocyte count 3±1.73; NL ratio = 1.27±1.1; Mean leukocyte count in non-malaria 9.5±1.2; Mean neutrophil count 2.7±1.11; Mean lymphocyte count 5.5±1.42; NL ratio = 0.49±0.78	UM = 104 SM = 48 SA = 15 HP = 13 HG = 3 CM = 17	Cell- Dyn 3000® (CD3000) instrument (Abbott, Santa Clara, California	368	152				30	216	Febrile
Hasona et al., 2016 [43]	Saudi Arabia (2014–2015)	All groups = 20–60		NA	*P*. *falciparum and P*. *vivax*	Mean leukocyte count in malaria - P.f. 4.21 ± 0.35; - P.v. 3.45 ± 0.12; Mean neutrophil count 2.9±0.04; Mean lymphocyte count 2.63±0.02; NL ratio = 1.1±2.0; Mean leukocyte count in non-malaria 6.94 ± 0.13; Mean neutrophil count 3.47±0.03; Mean lymphocyte count 2.78±0.02; NL ratio = 1.25±1.5	NA	SYSMX.KX-21n	120	6	24			20	90	Healthy
Hojo-Souza et al., 2015 [32]	Brazil (NA)	38.5 (19–61)	34.0 (22–37)	All groups = 48.7; Malaria = 27.3%; Non-malaria = 70%	*P*. *vivax*	Mean leukocyte count in malaria 5.5± 0.4; Mean neutrophil count 3.69±2.58; Mean lymphocyte count 1.51±1.23; NL ratio = 2.44±2.1; Mean leukocyte count in non-malaria 8.1 ± 0.5; Mean neutrophil count 5.27± 3.97; Mean lymphocyte count 2.65±2.22; NL ratio = 1.99±1.79	UM	ABX Pentra 90; Horiba Diagnostics, Kyoto, Japan	31		20			152	11	Healthy
Igbeneghu et al., 2011 [8]	Nigeria (NA)	31.9± 11.1	34.0± 12.1	All groups = 80.9; Malaria = 90.8%; Non-malaria = 70.7%	*P*. *vivax*, *P*. *malariae*, and mixed infections (3 cases)	Mean leukocyte count in malaria 4.68±1.4; Mean leukocyte count in non-malaria 5.38±2.1	UM	Coulter counter (STKS model)	668		136		2	138	527	Healthy
Jeremiah et al., 2007 [28]	Nigeria (2005–2006)	All groups = 1–8		All groups = 48.8%	*P*. *falciparum*	Mean leukocyte count in malaria 5.4±2.3; Mean leukocyte count in non-malaria 5.3±2.3	UM	Turk’s method	240	66				66	174	Healthy
Kayode et al., 2011 [29]	Nigeria (2010–2011)	All groups = 14–30		NA	*P*. *falciparum*	Mean leukocyte count in malaria 6.5±0.1; Mean neutrophil count 4.24±0.02; Mean lymphocyte count 2.26±0.02; NL ratio = 1.88±1.0; Mean leukocyte count in non-malaria 5.1±0.2; Mean neutrophil count 1.76±0.09; Mean lymphocyte count 3.93±0.06; NL ratio = 0.45±1.5	NA	WBC diluting fluid	40	30				30	10	Healthy
Kim et al., 2008 [4]	Republic of Korea (2000–2006)	26.1±11.1	24.5±3.7	All groups = 79.4; Malaria = 81.8%; Non-malaria = 76.9%	*P*. *vivax*	Mean leukocyte count in malaria 4.9±1.4; Mean leukocyte count in non-malaria 5.9±1.4	UM	Cell-Dyn 4000, Abbott diagnostics, USA	141		55			55	52	Healthy
Kimbi et al., 2013 [9]	Cameroon (2011)	All groups = 8.26±2.2		All groups = 47.8%	*P*. *falciparum*	Mean leukocyte count in malaria 5.1 ±2.5; Mean leukocyte count in non-malaria 6.3± 1.9	UM	Beckman Coulter counter (URIT 3000)	728	158				158	570	Febrile and healthy
Koltas et al., 2007 [35]	Turkey (2002–2004)	33.8±18.6	39±15	All groups = 61.5%	*P*. *vivax*	Mean leukocyte count in malaria 6.2 ±1.9; Mean leukocyte count in non-malaria 7.6± 2.2	NA	NA	142		90			90	52	Healthy
Kotepui et al., 2014 [13]	Thailand (2009)	24.5 (17–38)	16 (7–35)	All groups = 55.7%; Malaria = 60.3%; Non-malaria = 51.1%	*P*. *falciparum and P*. *vivax*	Mean leukocyte count in malaria 5.9±0.9; -P.f 6.0±1.0; -P.v 5.7±0.8; Mean neutrophil count 3.71±0.66; Mean lymphocyte count 1.35±0.37; NL ratio = 2.75±1.78; Mean leukocyte count in non-malaria 9.0±1.8; Mean neutrophil count 5.36±1.41; Mean lymphocyte count 2.39±0.57; NL ratio = 2.24±2.47	NA	BC-5200 Haematology Analyzer (Mindray, Nanshan, Shenzhen, China	4985	352	351			703	4282	Febrile
Maghendji-Nzondo et al., 2016 [44]	Gabon (2013–2014)	51.6±39.2	45.2±39	All groups = 52.3%; Malaria = 50%; Non-malaria = 54.5%	*P*. *falciparum*	Mean leukocyte count in malaria 5.6±4.1; Mean leukocyte count in non-malaria 11.4± 7.5	NA	Coulter STKS (STKS®, Coulter Corp, USA).	1129	530				530	1079	Febrile
Maghendji-Nzondo et al., 2016 [27]	Gabon (2011–2012)	63.4 ± 39.4	40.3 ± 37.1	All groups = 47%; Malaria = 50%; Non-malaria = 44%	*P*. *falciparum and* *P*. *malariae*	Mean leukocyte count in malaria 8.6 ± 6.4 Mean leukocyte count in non-malaria 10.8 ± 6.3	UM = 145 SM = 17 SA = 12 CM = 4 PT = 1	Coulter STKS (STKS®, Coulter Corp, USA).	940	158			4	162	778	Febrile
Okafor et al., 2016 [38]	South Africa (2012–2013)	NA		All groups = = 61.8%	*P*. *falciparum and* mixed infections (6 cases)	Mean leukocyte count in malaria 4.1 ± 0.46 (including mixed infections); Mean leukocyte count in non-malaria 5.6 ± 1.89	UM = 82 SM = 10 SA = 10	Sysmex XE 5000 Automated Haematology Analyser, (Sysmex, Canada)	92	6				6	86	Febrile
Ourives et al., 2015 [33]	Brazil (NA)	>18	35–55	All groups = = 44%; Malaria = NA; Non-malaria = 44%	*P*. *vivax*	Mean leukocyte count in malaria 7.6; Mean leukocyte count in control 6.0	NA	ABX PENTRA 90, (Horiba Diagnostic, Kyoto, Japan)	173		148			148	25	Healthy
Philipose CS and Umashankar T, 2016 [40]	India (2014)	36.1±17.1	48.3±19.2	NA	*P*. *falciparum*, *P*. *vivax and* mixed infections (18 cases)	Mean leukocyte count in malaria 6.3±3.1 (including mixed infections); Mean neutrophil count 3.96±2.98; Mean lymphocyte count 1.59±1.23; NL ratio = 2.49±2.42; Mean leukocyte count in control 9.5±5.7; Mean neutrophil count 6.56±5.52; Mean lymphocyte count 1.98±1.26; NL ratio = 3.33±4.38	NA	Beckmann Coulter® hematological analyzer	300	180	2			182	100	Febrile
Rodrigues-da-Silva et al., 2014 [45]	Brazil (2010)	All groups = 28.3 (22.5–40)		All groups = = 27%; Malaria = 27%; Non-malaria = NA	*P*. *falciparum* and *P*. *vivax*	Mean leukocyte count in malaria 5.1±0.8; - P.f = 4.9±0.8; - P.v = 5.2±0.8; Mean neutrophil count 3.35±0.63; Mean lymphocyte count 2.1±0.31; NL ratio = 1.6±2.2; Mean leukocyte count in controls; 6.5±0.6 Mean neutrophil count 3.85±0.5; Mean lymphocyte count 2.24±0.29; NL ratio = 1.72±1.72	UM	ABX PENTRA 90, (Horiba Diagnostic, Kyoto, Japan)	83	24	47			71	12	Healthy
Salih et al., 2018 [31]	Sudan (2015)	5.3±3.9	5.7±3	All groups = 59.4%	*P*. *falciparum*	Median leukocyte count in malaria 7.4 (5.2−9.5); Mean leukocyte count in severe malaria 8.98±2.06; Mean leukocyte count in uncomplicated malaria 7.33±1.09; Mean neutrophil count 4.63±1.17; Mean lymphocyte count 2.45±0.72 NL ratio = 1.89±1.63; Median leukocyte count in control 9.1 (5.3−12.4); Mean neutrophil count 3.45±1.15; Mean lymphocyte count 3.8±0.89; NL ratio = 0.91±1.29	UM = 63 SM = 67 CM = 10 CV = 18 SA = 9 HG = 1 HT = 7 JD = 6 HP = 9 MT1 = 8	Sysmex XN-9000; Hyogo, Japan	180	130				130	50	Healthy
Squire et al., 2016 [36]	Ghana (2012–2013)	4.9 (3.7–6.4)	3.96 (3.0–4.9)	All groups = 57.7%; Malaria = 64%; Non-malaria = 51.4%	*P*. *vivax*	Mean leukocyte count in malaria 9.5±1.6; Mean leukocyte count in severe malaria 8.91±0.8; Mean leukocyte count in uncomplicated malaria 10.1±2.8; Mean leukocyte count in control 8.9±0.9	UM = 24 SM = 81 SA = 2	Sysmex KX- 21 N, Japan	150	105				105	45	Febrile
Sumbele et al., 2017 [30]	Cameroon (2014)	All groups = 25.5		All groups = 49.6%	*P*. *falciparum*	Mean leukocyte count in malaria 8.9±3.3; Mean leukocyte count in control 8.4±3.4	UM = 124 SM = 1 SA = 1	URIT-3300 Automated Hematology Analyzer (Guilin Botest Medical Electronic Co. Ltd, PR China)	387	125				125	262	Febrile
Worku et al., 1997 [46]	Ethiopia (NA)	All groups = 27 (22–32)		All groups = 79.5%	*P*. *falciparum* and *P*. *vivax*	Mean leukocyte count in malaria 5.5±0.7; - P.f. = 4.9±0.6; - P.v. = 6.0±0.8; Mean leukocyte count in control -7.4±1.3	NA	NA	55	19	20			39	16	Healthy

UM = uncomplicated malaria, SM = severe malaria, SA = severe anemia, HP = hyperparasitemia, HG = hypoglycemia, CM = cerebral malaria, PT = prostration, HT = hypotension, CV = convulsion, JD = jaundice, MT1 = more than 1 complication.

### Malaria indicators

Twenty included studies (20/29, 69%) used only the microscopic method for detection of malaria parasites. Five studies used both microscopy and PCR [15, 32, 34, 39, 46]. Two studies used both microscopy and RDT [35, 38]. Two studies used three methods for malaria detection including microscopy, RDT, and PCR methods (S2 Table).

### Leukocyte indicators

Most of the leukocyte counts in the 29 studies were obtained using hematology analyzers from different manufacturers. Six studies used a Sysmex [15, 31, 36, 38, 41, 43]. Three studies used a Cell-Dyn [4, 5, 7]. Three studies used an ABX [32, 39, 45]. Three studies used a Beckman-Coulter [6, 9, 40]. Two studies used a Coulter STKS [27, 44]. Other studies used a Coulter counter [8], BC-5200 Haematology Analyzer [13], and URIT-3300 [30]. Two studies used Turk’s method, which is a traditional protocol for leukocyte counting using a hemocytometer [28, 29].

### Quality of included studies

All 29 studies included in the present study were rated with a score according to the NOS guidelines. Overall, twenty-eight studies were rated “good” with a maximum of 9 stars, and one study was rated “medium” with 8 stars because there was no clear definition of the non-malaria group reported by the authors. The rating details are provided in Table 2.

**Table 2:** Quality of the included studies.

No.	Reference	Selection				Compatibility	Exposure			Quality of included studies
		Is the Case Definition Adequate?	Representativeness of the Cases	Selection of Non-malaria	Definition of Non-malaria		Ascertainment of Exposure	Same method of ascertainment for cases and non-malaria	Nonresponse Rate	
1.	Adam et al. [41]	**✵**	**✵**	**✵**	**✵**	**✵✵**	**✵**	**✵**	**✵**	Good
2.	Ansart et al.[37]	**✵**	**✵**	**✵**	**✵**	**✵✵**	**✵**	**✵**	**✵**	Good
3.	Anwar et al. [42]	**✵**	**✵**	**✵**	**✵**	**✵✵**	**✵**	**✵**	**✵**	Good
4.	Awoke N and Arota A [5]	**✵**	**✵**	**✵**	**✵**	**✵✵**	**✵**	**✵**	**✵**	Good
5.	Chaves et al. [15]	**✵**	**✵**	**✵**	**✵**	**✵✵**	**✵**	**✵**	**✵**	Good
6.	Erhart et al [6]	**✵**	**✵**	**✵**	**✵**	**✵✵**	**✵**	**✵**	**✵**	Good
7.	Frimpong et al. [26]	**✵**	**✵**	**✵**	**✵**	**✵✵**	**✵**	**✵**	**✵**	Good
8.	Goncalves et al. [39]	**✵**	**✵**	**✵**	**✵**	**✵✵**	**✵**	**✵**	**✵**	Good
9.	González et al. [34]	**✵**	**✵**	**✵**	**✵**	**✵✵**	**✵**	**✵**	**✵**	Good
10.	Hänscheid et al. [7]	**✵**	**✵**	**✵**	**✵**	**✵✵**	**✵**	**✵**	**✵**	Good
11.	Hasona et al. [43]	**✵**	**✵**	**✵**	**✵**	**✵✵**	**✵**	**✵**	**✵**	Good
12.	Hojo-Souza et al. [32]	**✵**	**✵**	**✵**	**✵**	**✵✵**	**✵**	**✵**	**✵**	Good
13.	Igbeneghu et al. [8]	**✵**	**✵**	**✵**	**✵**	**✵✵**	**✵**	**✵**	**✵**	Good
14.	Jeremiah et al. [28]	**✵**	**✵**	**✵**	**✵**	**✵✵**	**✵**	**✵**	**✵**	Good
15.	Kayode et al. [29]	**✵**	**✵**	**✵**	**✵**	**✵✵**	**✵**	**✵**	**✵**	Good
16.	Kim et al. [4]	**✵**	**✵**	**✵**	**✵**	**✵✵**	**✵**	**✵**	**✵**	Good
17.	Kimbi et al. [9]	**✵**	**✵**	**✵**	**✵**	**✵✵**	**✵**	**✵**	**✵**	Good
18.	Koltas et al. [35]	**✵**	**✵**	**✵**	**✵**	**✵✵**	**✵**	**✵**	**✵**	Good
19.	Kotepui et al. [13]	**✵**	**✵**	**✵**	**✵**	**✵✵**	**✵**	**✵**	**✵**	Good
20.	Maghendji-Nzondo et al. [44]	**✵**	**✵**	**✵**	**✵**	**✵✵**	**✵**	**✵**	**✵**	Good
21.	Maghendji-Nzondo et al. [27]]	**✵**	**✵**	**✵**	**✵**	**✵✵**	**✵**	**✵**	**✵**	Good
22.	Okafor et al. [38]	**✵**	**✵**	**✵**		**✵✵**	**✵**	**✵**	**✵**	Medium
23.	Ourives et al. [33]	**✵**	**✵**	**✵**	**✵**	**✵✵**	**✵**	**✵**	**✵**	Good
24.	Philipose CS and Umashankar T [40]	**✵**	**✵**	**✵**	**✵**	**✵✵**	**✵**	**✵**	**✵**	Good
25.	Rodrigues-da-Silva et al. [45]	**✵**	**✵**	**✵**	**✵**	**✵✵**	**✵**	**✵**	**✵**	Good
26.	Salih et al. [31]	**✵**	**✵**	**✵**	**✵**	**✵✵**	**✵**	**✵**	**✵**	Good
27.	Squire et al. [36]	**✵**	**✵**	**✵**	**✵**	**✵✵**	**✵**	**✵**	**✵**	Good
28.	Sumbele et al. [30]	**✵**	**✵**	**✵**	**✵**	**✵✵**	**✵**	**✵**	**✵**	Good
29.	Worku et al. [46]	**✵**	**✵**	**✵**	**✵**	**✵✵**	**✵**	**✵**	**✵**	Good

### Meta-analysis

**Fig 2:**
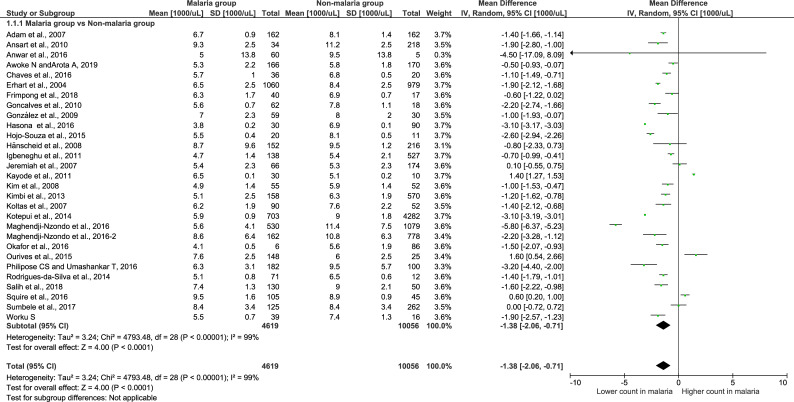
Forest plot of the total leukocyte count among included studies. Forest plot showing the total leukocyte count in the malaria and non-malaria groups. [1000/μL] refers to 1000 per microliter; “IV" in "IV, Random” refers to Inverse variance; "Total" in top row refers to number of patients included; A green square in the horizontal line refers to the mean difference for each of included study.

A meta-analysis of the leukocyte count in the malaria and non-malaria groups was conducted to examine the statistical significance and mean difference across the 29 studies. The analysis demonstrated that there were significantly lower leukocyte counts in patients in the malaria group than in those in the non-malaria group (Z = 4.0, P-value < 0.00001, mean difference = -1.38, 95% CI = -2.06-(-0.71)). Only 2 of the 29 included studies presented a significantly higher total leukocyte count in the malaria group than in the non-malaria group [29, 33] (Fig 2). Five out of twenty-nine studies presented total leukocyte counts that were not significantly different in the malaria group and the non-malaria group [7, 26, 28, 30, 42].

### Data heterogeneity

**Fig 3:**
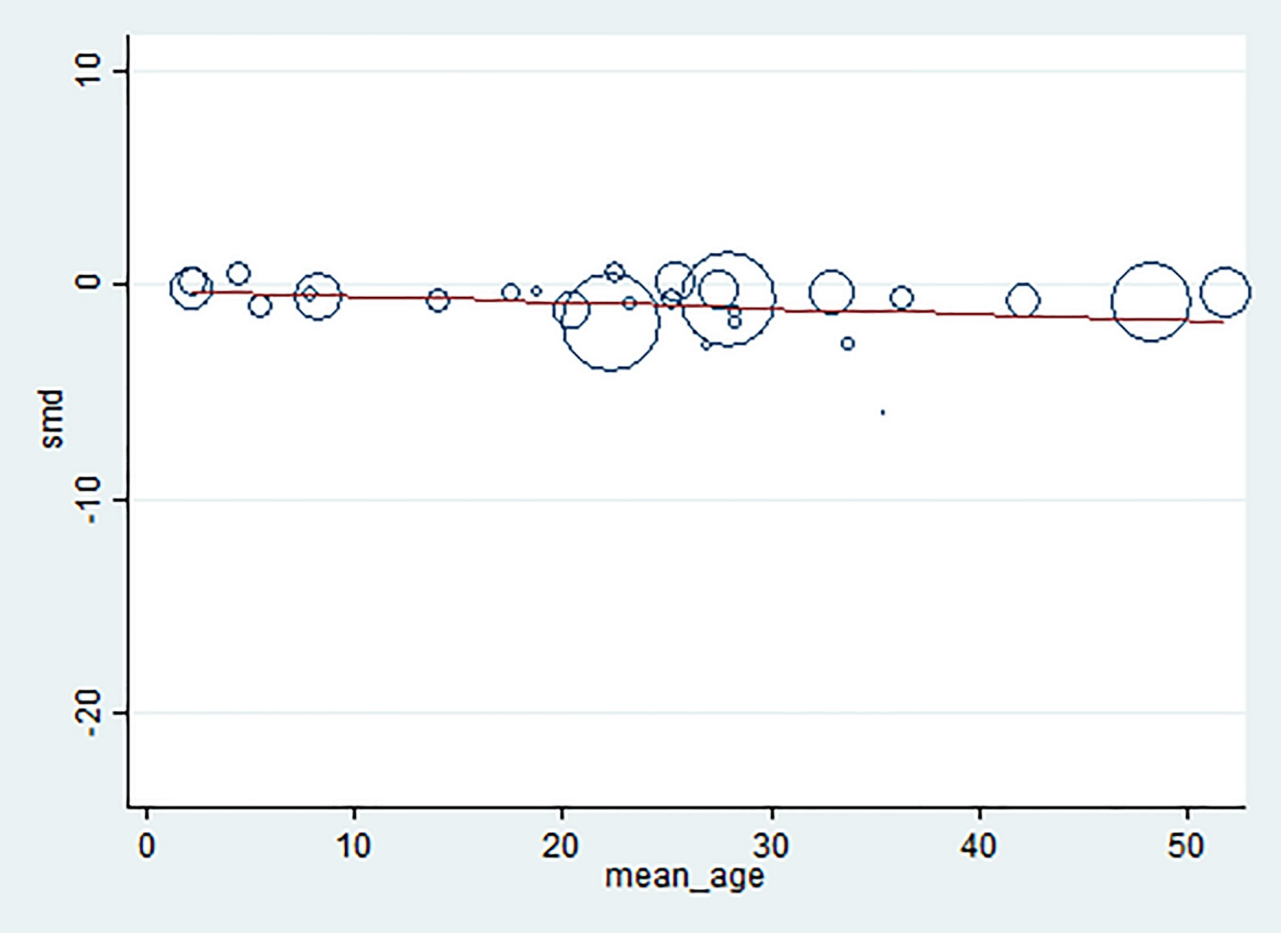
Meta-regression graph of mean age as a covariate.

The present study used a forest plot, heterogeneity test (Cochran’s Q), and the degree of heterogeneity (I^2^) to test whether the included studies had heterogeneity. The results from the forest plot indicated that the standard deviations overlapped among the included studies. Cochran’s Q indicated that the results were significant (P-value < 0.000001, Chi^2^ = 4794.7, df = 28, Tau^2^ = 3.23) with an I^2^ of 99%. A meta-regression with mean age as a covariate was performed to determine if age was the source of heterogeneity or whether it modified the outcome. Meta-regression using STATA software indicated that mean age was not the source of heterogeneity and it did not modify the outcome (P-value = 0.48, percent of residual variation (I^2^) = 97.9% (Table 3, Fig 3).

**Table 3:** Meta-regression analysis of mean age.

SMD	Coefficient	Standard error	t-statistic	P-value	95% CI
Mean age	-0.03	0.04	-0.72	0.48	-0.11–0.05
Constant	-0.25	1.02	-0.25	0.81	-2.35–1.84

* SMD: The standardized mean difference.

### Meta-analysis of leukocyte differential counts

**Fig 4:**
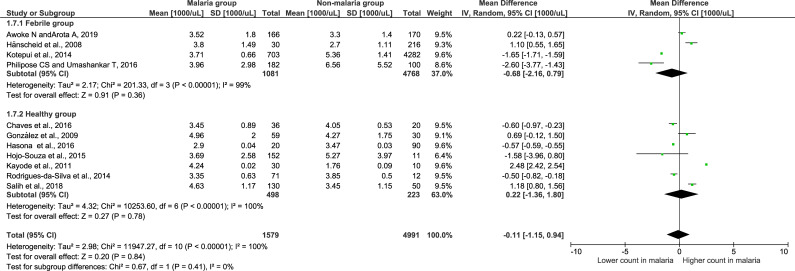
Forest plot of the neutrophil count among included studies. Forest plot showing the neutrophil count in the malaria and non-malaria groups. [1000/μL] refers to 1000 per microliter; “IV" in "IV, Random” refers to Inverse variance; "Total" in top row refers to number of patients included; A green square in the horizontal line refers to the mean difference for each of included study.

**Fig 5:**
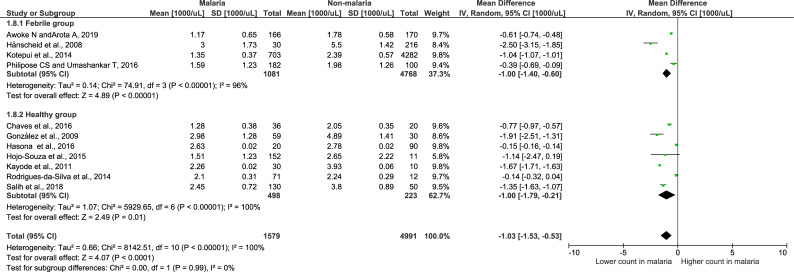
Forest plot of the lymphocyte count among included studies. Forest plot showing the lymphocyte count in the malaria and non-malaria groups. [1000/μL] refers to 1000 per microliter; “IV" in "IV, Random” refers to Inverse variance; "Total" in top row refers to number of patients included; A green square in the horizontal line refers to the mean difference for each of included study.

**Fig 6:**
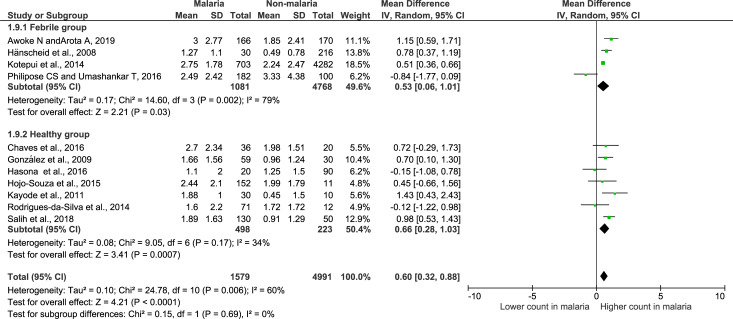
Forest plot of the NL ratio among included studies. Forest plot showing the NL ratio in the malaria and non-malaria groups. [1000/μL] refers to 1000 per microliter; “IV" in "IV, Random” refers to Inverse variance; "Total" in top row refers to number of patients included; A green square in the horizontal line refers to the mean difference for each of included study.

Meta-analysis of leukocyte differential counts was available in 11 studies and was also analyzed. For neutrophil counts, the meta-analysis showed no difference in neutrophil counts between the malaria and non-malaria groups (P value = 0.84, mean difference = -0.11, 95% CI = -1.15–0.94) (Fig 4). For lymphocyte counts, the results showed that the malaria group had a significantly lower lymphocyte count than the non-malaria group (P value <0.0001, mean difference = -1.03, 95% CI = -1.53-(-0.53)) (Fig 5). For the NLR, the results showed that the NLR was higher in the malaria group than in the non-malaria group (P value <0.0001, mean difference = 0.6, 95% CI = 0.32–0.88) (Fig 6). The difference of leukocyte count between severe and non-severe malaria group was also analyzed. The results demonstrated no significant difference of the total leukocyte count between severe and non-severe group (P value = 0.37).

### Subgroup analysis of the febrile and healthy groups

**Fig 7:**
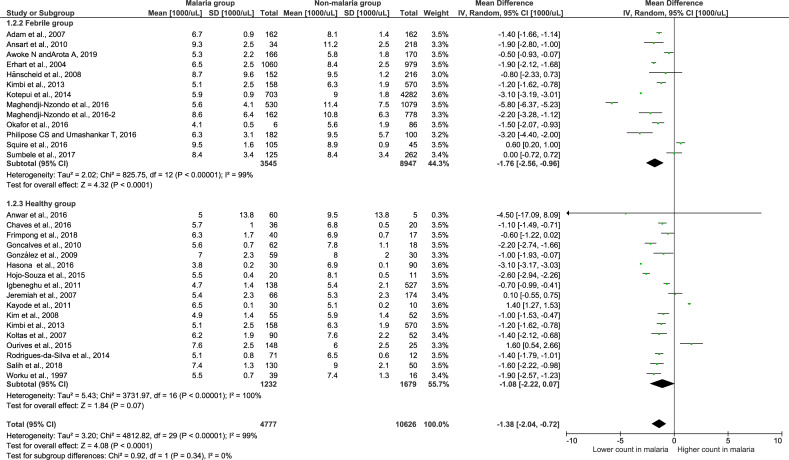
Subgroup analysis of the total leukocyte count. Forest plot of the subgroup analysis showing the total leukocyte count in malaria compared with the febrile and healthy groups. [1000/μL] refers to 1000 per microliter; “IV" in "IV, Random” refers to Inverse variance; "Total" in top row refers to number of patients included; A green square in the horizontal line refers to the mean difference for each of included study.

To determine whether using febrile and healthy controls impacts the differences in total leukocyte counts, a subgroup analysis of the non-malaria groups was conducted (Fig 7). The results showed that 13 studies using febrile controls demonstrated a significantly lower leukocyte count in the malaria group than in the non-malaria group (Z = 1.33, P-value < 0.0001, mean difference = -1.76, 95% CI = -2.56-(-0.96), I^2^ = 99%). Interestingly, seventeen studies using healthy participants as a control group demonstrated no significance in the leukocyte count among the malaria and healthy groups (Z = 1.33, P-value = 0.07, mean difference = -1.07, 95% CI = -2.22–0.07, I^2^ = 100%). The subgroup analysis showed no significant difference between subgroups (P-value = 0.34), demonstrating that the subgroup (febrile or healthy) was not the source of the heterogeneity or that it could not explain the heterogeneity among the 29 included studies.

### Analysis of *P*. *falciparum* using febrile and healthy groups

**Fig 8:**
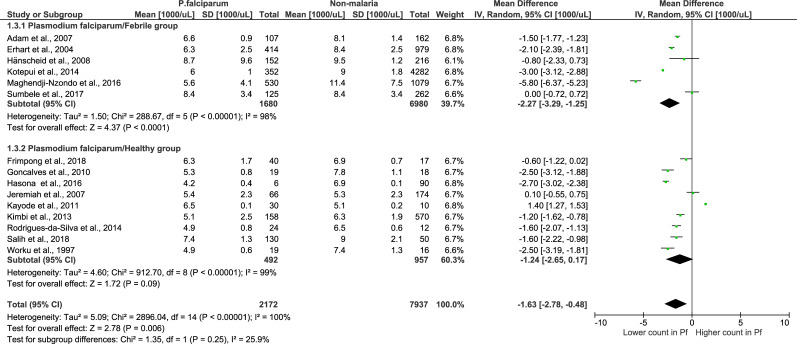
Subgroup analysis of *P*. *falciparum*. Forest plot of subgroup analysis showing the total leukocyte count in the *P*. *falciparum* group compared to the febrile and healthy groups. [1000/μL] refers to 1000 per microliter; “IV" in "IV, Random” refers to Inverse variance; "Total" in top row refers to number of patients included; A green square in the horizontal line refers to the mean difference for each of included study.

When using febrile groups, the analysis of 6 studies with *P*. *falciparum* monoinfection demonstrated that there was a significantly lower total leukocyte count in the *P*. *falciparum* monoinfection group than in the febrile group (Z = 4.37, P-value < 0.0001, mean difference = -2.27, 95% CI = -3.29-(-1.25), I^2^ = 98%) (Fig 8). Whereas, in studies using healthy groups, the analysis of 9 studies with *P*. *falciparum* monoinfection demonstrated that there was no significant difference in leukocyte counts in the *P*. *falciparum* monoinfection group and the healthy group (Z = 1.72, P-value = 0.09, mean difference = -1.24, 95% CI = -2.65–0.17, I^2^ = 99%). The subgroup analysis showed that there was no significant difference between subgroups (P-value = 0.25), demonstrating that the subgroup (febrile or healthy) was not the source of the heterogeneity or that it could not explain the heterogeneity among the 9 included studies.

### Analysis of *P*. *vivax* using febrile and healthy controls

**Fig 9:**
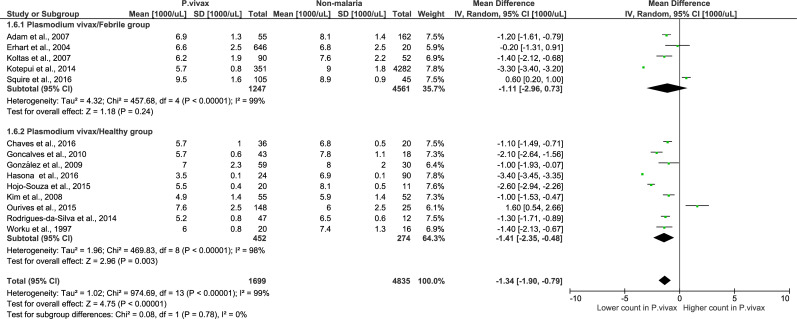
Subgroup analysis of *P*. *vivax*. Forest plot of subgroup analysis showing the total leukocyte count in *P*. *vivax* compared to febrile and healthy groups. [1000/μL] refers to 1000 per microliter; “IV" in "IV, Random” refers to Inverse variance; "Total" in top row refers to number of patients included; A green square in the horizontal line refers to the mean difference for each of included study.

For *P*. *vivax* monoinfection, when using a febrile group, the analysis using 5 studies with *P*. *vivax* monoinfection demonstrated that there was no significant difference in total leukocyte counts in the *P*. *vivax* monoinfection group compared to the febrile group (Z = 1.18, P-value = 0.24, mean difference = -1.11, 95% CI = -2.96–0.73, I^2^ = 99%). When using the healthy group as a control, the analysis using 9 studies with *P*. *vivax* monoinfection demonstrated that there was a significantly lower total leukocyte count in the *P*. *vivax* monoinfection group than in the healthy group (Z = 2.96, P-value = 0.003, mean difference = -1.14, 95% CI = -2.35–0.48, I^2^ = 98%). The subgroup analysis showed that there was no significant difference between the subgroups (P-value = = 0.78) (Fig 9), demonstrating that the subgroup (febrile or healthy) was not the source of the heterogeneity or that it could not explain the heterogeneity among the 5 included studies.

### Publication bias

**Fig 10:**
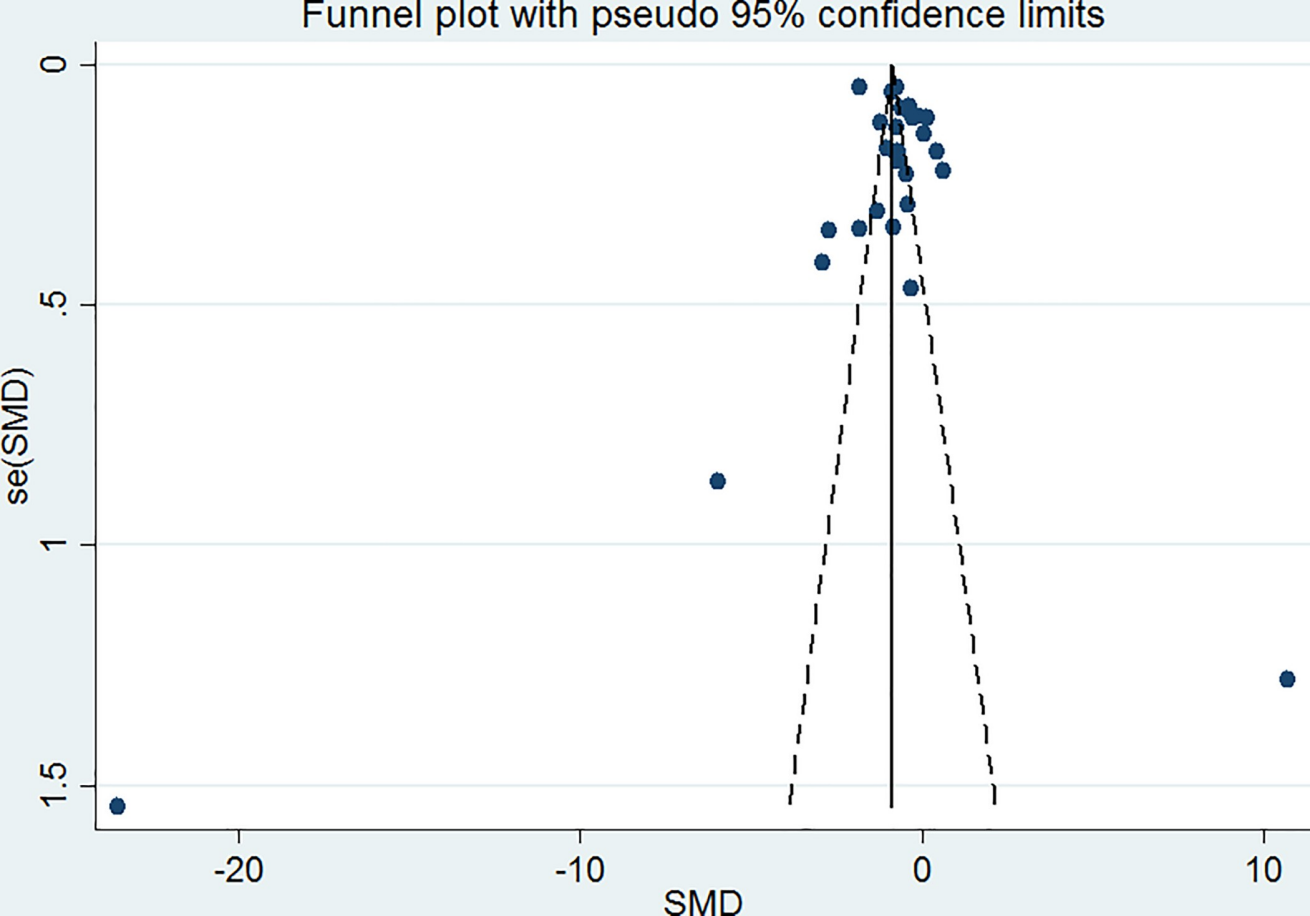
Funnel plot. Funnel plot showing publication bias among the included studies.

Funnel plot analysis generated a symmetrical funnel plot (Fig 10). The symmetry of the funnel plot was assessed by Egger’s test, which indicated that no small-study effects were found by using linear regression analysis (P-value = 0.497, slope coefficient = -0.99). The symmetrical funnel plot indicated that there was no publication bias.

## Discussion

This systematic review and meta-analysis described the pooled mean difference of the total leukocyte count of our study. The results demonstrated that there was a lower total leukocyte count in the malaria group than in the non-malaria groups. The lower total leukocyte count in this meta-analysis might be explained by the localization of leukocytes away from the peripheral circulation, such as in the spleen, at the sites of infection, or in other peripheral pools, resulting in a low number of total leukocytes detected in the circulation [12, 47]. A previous study suggested that the alteration of immune cells in the peripheral blood was also the cause of leukopenia [48]. Immunity against malaria parasite invasion and the infection of red blood cells is very high during the liver stage or exoerythrocytic stage compared to the erythrocytic stage, and major immune responses for these two stages involve CD8^+^ T cells and antibodies, respectively [49]. Another possible immune mechanism against malaria infection involves interleukin 12 (IL-12), which is involved in the pathogenesis of malarial pancytopenia, the pathogenesis of low total leukocytes, red blood cells, and platelet production from the bone marrow [50]. One study suggested that the glycosylphosphatidylinositol antigen of malaria induces monocyte and macrophage activation, resulting in the release of proinflammatory cytokines, such as tumor necrosis factor-α (TNF-α) and IL-1α, and the phagocytosis of both infected red blood cells and leukocytes [51]. Several studies have reported that TNF, IL-12, IL-10, and other cytokines can suppress the production of leukocytes from bone marrow by inhibiting hemopoietic growth factors or stimulating macrophages to release cytotoxic chemicals, causing damage to hemopoietic cells [52–55].

The differences in leukocyte differential counts were also assessed in the present study. Among the 11 included studies that reported differential counts, the results demonstrated that the absolute lymphocyte count and NLR were significantly altered during malaria infection. The absolute lymphocyte counts of the malaria group were low, and the NLR was high. These results were consistent with our previous study demonstrating that neutrophil and lymphocyte counts were the most important leukocytic changes associated with malaria infection [13]. Our previous study also demonstrated that there was a significantly higher NLR in the malaria-infected group than in the noninfected group [13]. Moreover, the NLR was found to be correlated with malaria parasitemia, and it was inferior to CRP as a marker for severe imported malaria [56]. The reduction in lymphocyte counts in the present study may be due to the redistribution of lymphocytes, with sequestration in the spleen of malaria-infected patients [50].

Heterogeneity was observed in the 29 articles included in the present study. Although we used covariates or confounders such as age groups and subgroup analyses of *Plasmodium* species and febrile/healthy groups, they did not reduce the degree of heterogeneity (I^2^) in the included studies. Regarding the test for publication bias by Egger’s test (which might determine publication bias as the source of heterogeneity), no publication bias was found among the 29 studies. It could be concluded that publication bias was not the underlying cause of data heterogeneity in the articles included in the present study.

In our systematic review and meta-analysis, the non-malaria groups included both febrile patients who were negative for malaria parasites and healthy individuals who were located in the same areas as the malaria group. The results demonstrated that there was a lower total leukocyte count in the malaria group than in the non-malaria group. However, a subgroup analysis of the febrile and healthy groups indicated that there was a significantly lower total leukocyte count in the malaria group than in the febrile group, while a subgroup analysis of the seventeen studies using the healthy group as a control demonstrated that there was no significant decrease in the total leukocyte count in the malaria group. The total leukocyte count in the malaria group was significantly different from that in the febrile group. Therefore, it is crucial for researchers to report the total leukocyte count in malaria patients compared to that in febrile control individuals rather than compared with healthy individuals; a low leukocyte count is likely to be a useful indicator to aid in malaria diagnosis in malaria-endemic areas.

To confirm whether each *Plasmodium* species differentially impacted the total leukocyte count, a subgroup analysis of the total leukocyte count in each *Plasmodium* spp. was conducted. The mean difference indicated that there was a significant reduction in the total leukocyte count in patients with *P*. *falciparum* and *P*. *vivax* monoinfection compared to that in patients in the non-malaria group. Compared with the febrile group, the *P*. *falciparum* monoinfection group had a lower total leukocyte count, while the *P*. *vivax* monoinfection group showed no difference in the total leukocyte count. Compared with the healthy group, the *P*. *falciparum* monoinfection group did not show a difference in the total leukocyte count, while the *P*. *vivax* monoinfection group had a lower total leukocyte count. We did not analyze the difference in the total leukocyte count between *P*. *falciparum* and *P*. *vivax* because the included studies did not report total leukocyte counts separately for each species, but some previous studies indicated that there was a significantly lower total leukocyte count in patients with *P*. *falciparum* monoinfection than in those with *P*. *vivax* monoinfection [6, 39, 45, 46].

The meta-analysis demonstrated that two studies in Nigeria (2010–2011) and Brazil [29, 33] presented an increase in the total leukocyte count in malaria patients compared to non-malaria patients. The study by Kayode et al. suggested that the increased total leukocyte count was due to the increased mobilization of leukocytes from the bone marrow to the bloodstream to fight against malarial parasites [29]. Another study indicated that there was no significant difference in the total leukocyte count [33]. Both studies used healthy control groups for the comparison of the total leukocyte count in the malaria group. Interestingly, the latter study had results consistent with the present study, indicating that there was no significant difference in the total leukocyte count in malaria and healthy groups [33]. All of these reports did not indicate the number of days of fever before admission. Our previous study indicated that there were lower neutrophil/monocyte and higher lymphocyte counts when comparing patients with fever ≤ 3 days and patients with fever > 3 days (P-value < 0.05). However, the total leukocyte count did not change during the 3 days before admission [57]. The association of the number of days of fever with the leukocyte count might be explained by a study among native Dutch volunteers who were bitten by infected mosquitoes in a Controlled Human Malaria Infection (CHMI) model, which demonstrated an increase in the peripheral total leukocyte count during malarial infection in the liver stage; however, a subsequent decrease occurred when the parasites appeared in the peripheral blood [10]. The mechanism of leukocytosis might involve the infection by malaria parasites in the bone marrow (which would inhibit the release of leukocytes), increases in proinflammatory cytokines in the peripheral blood, or febrile paroxysms [50].

A study among children in Africa indicated that a high leukocyte count was associated with a great risk for death [17]. The cause of death might be delayed diagnosis, as a previous study indicated that there was an initial increase in peripheral total leukocyte count during liver-stage infection and a significantly lower total leukocyte count during the blood stage [10]. Here, we present gathered data that support evidence of total leukocyte count as an early detection tool to screen malarial infection in conjunction with other malarial confirmation tests, such as in routine malaria diagnosis in malaria-endemic areas. Further, these meta-analytic results provide clinicians with a rationale for instituting specific initiatives and early interventions in malaria-endemic areas, which may decrease mortality and provide less complicated and possibly more economical management of potential *Plasmodium-*related patients, even before the actual confirmation of *Plasmodium* infection. A higher total leukocyte count might indicate the initial stage of malarial infection in the liver, while a low total leukocyte count could be an indicator of the progression of malarial infection during the blood stage.

### Summary of evidence

In summary, our recommendations for further work include using febrile individuals as a control group to provide data on the differences in the total leukocyte count between the malaria group and non-malaria groups. This would allow for a precise understanding of malarial infection-related outcomes and could identify important predictors of the prognosis of malaria patients. The standardization of both malaria detection techniques and total leukocyte indicators would ensure improved comparability between studies as well as a common approach for reporting results, which should minimize heterogeneity and allow the calculation of precise effect measures in a model.

### Limitations

The present study had several limitations. First, the heterogeneity, which was not due to publication bias and other confounders that were already tested in this meta-regression analysis, was a limitation. Some of the published data could not be retrieved because the full-text manuscripts were not available. Second, specific leukocyte changes might vary with the level of malaria endemicity, background hemoglobinopathy, nutritional status, demographic factors, and malaria immunity [58], which could not be ruled out from this study based on the exclusion criteria. Third, two included studies measured the total leukocyte count using manual methods [28, 29], which might result in unreliable total leukocyte counts during meta-analysis and impact the mean difference. These two studies demonstrated that there was a higher mean leukocyte count in the malaria group than in the non-malaria groups. Fourth, the results of the present study need to be carefully interpreted, as the specific diagnoses in the febrile groups were not reported in the included studies. Such information may be highly relevant, such as bacterial or viral diseases that are likely to cause leukocytosis or dengue, which is likely to cause leukopenia.

## Conclusion

The present systematic review demonstrated that the total leukocyte count was a hematological parameter that is affected by malarial infection before treatment. Despite the heterogeneity of the included studies, a significant reduction in the total leukocyte count in malaria patients is a concern. We recommend including febrile individuals in the non-malaria group to provide data on the differences in total leukocyte counts in the malaria groups and non-malaria groups. Future studies must aim to understand the effects of *Plasmodium* infection on each specific leukocyte subpopulation, as well as interventions that cause total leukocyte reductions during follow-up or post-treatment outcomes in malaria-endemic settings.

## Supporting information

S1 Checklist PRISMA statement for reporting systematic reviews and meta-analyses. (DOC)

S1 Table Search strategy. (DOCX)

S2 Table Detection methods for malaria diagnosis. (DOCX)
