# Supplementary material for: Reduction in total leukocytes in malaria patients compared to febrile controls: A systematic review and meta-analysis
Source: PLoS One. 2020 Jun 23;15(6):e0233913. doi: 10.1371/journal.pone.0233913 (PMC7310711; doi:10.1371/journal.pone.0233913)
Supplement: S1 Table — (DOCX) [file pone.0233913.s002.docx]

**Supplementary Table 2. Search details for the three databases**

| ("malaria" OR "plasmodium") AND ("leukocyte" OR "white blood cell") |
| --- |
