# Supplementary material for: Reduction in total leukocytes in malaria patients compared to febrile controls: A systematic review and meta-analysis
Source: PLoS One. 2020 Jun 23;15(6):e0233913. doi: 10.1371/journal.pone.0233913 (PMC7310711; doi:10.1371/journal.pone.0233913)
Supplement: S2 Table — (DOCX) [file pone.0233913.s003.docx]

**Supplementary Table 1**. Detection methods for malaria diagnosis

| **No.** | **Reference** | **Detection methods** |
| --- | --- | --- |
| 1. | Adam et al., 2017 | Microscopy |
| 2. | Ansart et al., 2010 | Microscopy |
| 3. | Anwar et al., 2016 | Microscopy |
| 4. | Awoke N and  Arota A, 2019 | Microscopy |
| 5. | Chaves et al., 2016 | Microscopy, PCR |
| 6. | Erhart et al., 2004 | Microscopy |
| 7. | Frimpong et al., 2018 | Microscopy |
| 8. | Goncalves et al., 2010 | Microscopy, PCR |
| 9. | González et al., 2009 | Microscopy, PCR |
| 10. | Hänscheid et al., 2008 | Microscopy |
| 11. | Hasona et al., 2016 | Microscopy |
| 12. | Hojo-Souza et al., 2015 | Microscopy, PCR |
| 13. | Igbeneghu et al., 2011 | Microscopy |
| 14. | Jeremiah et al., 2007 | Microscopy |
| 15. | Kayode et al., 2011 | Microscopy |
| 16. | Kim et al., 2008 | Microscopy |
| 17. | Kimbi et al., 2013 | Microscopy |
| 18. | Koltas et al., 2007 | Microscopy, RDT |
| 19. | Kotepui et al., 2014 | Microscopy |
| 20. | Maghendji-Nzondo et al., 2016 | Microscopy, RDT, PCR |
| 21. | Maghendji-Nzondo et al., 2016 | Microscopy, RDT, PCR |
| 22. | Okafor et al., 2016 | Microscopy, RDT |
| 23. | Ourives et al., 2015 | Microscopy |
| 24. | Philipose CS and Umashankar T, 2016 | Microscopy |
| 25. | Rodrigues-da-Silva et al., 2014 | Microscopy |
| 26. | Salih et al., 2018 | Microscopy |
| 27. | Squire et al., 2016 | Microscopy |
| 28. | Sumbele et al., 2017 | Microscopy |
| 29. | Worku et al., 1997 | Microscopy, PCR |
